# Measuring the impact and distress of osteoarthritis from the patients' perspective

**DOI:** 10.1186/1477-7525-7-37

**Published:** 2009-04-29

**Authors:** Julie F Pallant, Anne-Maree Keenan, Roseanne Misajon, Philip G Conaghan, Alan Tennant

**Affiliations:** 1 School of Rural Health, University of Melbourne, 49 Graham St, Shepparton, Victoria, 3630, Australia; 2 Section of Musculoskeletal Disease, Leeds Institute of Molecular Medicine, University of Leeds, 2nd Floor, Chapel Allerton Hospital, Chapeltown Road, Leeds LS7 4SA, UK; 3 School of Political and Social Inquiry, Monash University, 900 Dandenong Road, Caulfield East, Victoria, 3145, Australia; 4 Department of Rehabilitation Medicine, University of Leeds, D Floor, Martin Wing, The General Infirmary at Leeds, Great George Street, Leeds LS1 3EX, England, UK

## Abstract

**Background:**

To assess the internal construct validity of the Perceived Impact of Problem Profile (PIPP), a patient based outcome measure based on the International Classification of Functioning, Disability and Health (ICF), which assesses impact and distress, in an osteoarthritis (OA) cohort.

**Methods:**

A questionnaire comprising the 23-item PIPP, which assesses five domains (mobility, participation, self care, psychological well being and relationships), the Western Ontario McMasters University Osteoarthritis Index (WOMAC), the General Well-Being Index (GWBI), and the Hospital Anxiety and Depression Scale (HADS) was posted to people with clinician diagnosed OA. Assessment of the internal construct validity of the PIPP was undertaken using Rasch analysis performed with RUMM2020 software and concurrent validity through comparator measures.

**Results:**

Two hundred and fifty-nine participants with OA responded. Analysis of the five individual domains of the PIPP indicated that there was good fit to the Rasch model, with high person separation reliability. One item required removal from the Mobility subscale and the Participation subscale. There were strong correlations between the PIPP Mobility scores and the WOMAC disability and pain subscales (rho = .73 and rho = .68), and between the PIPP Psychological well-being and HADS Depression (rho = .71) and GWBI (rho = -.69). High inter-correlations between the impact and distress subscales for each domain (range rho = .85 to .96), suggested redundancy of the latter.

**Conclusion:**

This study demonstrates that the PIPP has good psychometric properties in an OA population. The PIPP, using just the impact subscales, provides a brief, reliable and valid means of assessing the impact of OA from the individual's perspective and operationalizing the bio-psychosocial model by the application of a single multi-domain questionnaire.

## Background

Osteoarthritis (OA) is the most common cause of musculoskeletal pain [1] and is one of the ten most disabling diseases in developed countries [2]. Worldwide estimates indicate that one in ten men and one in five women aged over 60 have symptomatic OA [2]. Those with arthritis report significant pain and functional limitations [3,4], and are more likely to perceive themselves as mentally and physically unhealthy [5] and they represent a considerable burden on health care expenditure [6-8]. While OA of the hip and knee account for the largest component of the burden of the disease [9,10], the wider impact and distress of living with OA has generally been poorly described in the literature. While several outcome-based tools have been developed to evaluate OA, such as the WOMAC [11] and the Lequesne Index [12] they predominately measure pain and function, which has been the focus of the majority of OA research to date. However, increasing emphasis is being placed upon the socioeconomic and psychosocial issues associated with OA [13], attempting to measure the constructs patients consider to be important [14-16] and widen the understanding of the consequences of disease to the broader bio-psychosocial model [17].

One recently developed instrument attempts to capture some of the key elements of such a model, incorporating elements of the International Classification of Functioning, Disability and Health (ICF) [17], as well as key psychological components such as well-being. The Perceived Impact of Problem Profile (PIPP) [18] was developed to provide a generic research and clinical measurement tool to assess both the impact and distress of health conditions from the individuals' perspective. It contains a set of standardized domains (Self-care, Mobility, Participation, Relationships, and Psychological Well-being) to allow comparison of scores across patient groups and within individuals over multiple time points. The selection of domains was guided in part by the ICF, together with a review of existing measures, and a series of qualitative interviews. Consequently, items were selected to assess the impact and distress of the health condition on Activities and Participation (ICF Chapters 4 to 9) [19], and on the individual's psychological well-being (including independence and autonomy), an area not yet well addressed in the ICF.

The psychometric properties of the PIPP were previously assessed using Rasch analysis in a sample of those with locomotor disorders [18]. All subscales recorded adequate person separation reliability and no evidence of item bias for sex, age, educational achievement or rural versus urban residence. Preliminary validity testing using the individual items from the EQ5D provided support for the PIPP subscales [18]. Further validation across a range of health conditions and patient groups was recommended.

The aim of the current study was to extend the validity testing of the PIP by (a) assessing the psychometric properties of the PIPP in a sample of patients with OA and (b) exploring the impact of OA on patients' lives across each of the ICF domains represented on the PIPP.

## Methods

### Participants

A postal survey was sent to 635 people in Leeds (UK) with OA. Participants were included if they were attending either the primary (Leeds Musculoskeletal Service) or secondary care (Rheumatology or Orthopaedics Clinics) services and had a positive diagnosis of OA. Participants with hip, hand and knee OA were included if they fulfilled the ARC Criteria for the Diagnosis of OA [20-22]; in the absence of any such criteria for foot OA, patients were included if they had symptomatic pain that was confirmed by clinician diagnosis of OA. Non-responders were sent two reminder letters, after which they were deemed to not wish to be part of the study.

### Materials

Participants were sent a questionnaire pack which included demographic information, (e.g. age, gender), co-morbidities (self reported, as diagnosed by a doctor or health professional and reported by the participant), the site(s) of OA, and the following validated measures:

• The Perceived Impact of Problem Profile (PIPP) [18] consists of 23 items, measuring five domains (Mobility, Self-care, Relationships, Participation and Psychological Well-being). It was developed as a generic research and clinical tool to assess the impact and distress associated with a health condition. For each item, respondents were asked to rate (a) 'how much impact has your current health problems had on [item of function or activity]'; and (b) 'How much distress has been caused by the impact of your health problem on [same item of function or activity]'. The 6-point scale was anchored by 'no impact' and 'extreme impact' for the Impact scales and by 'no distress' and 'extreme distress' for the Distress scales.

• The Western Ontario McMasters University Osteoarthritis Index (WOMAC) is a disease specific questionnaire where statements are rated on a 0 (no problem) to 4 (extreme problem) over three domains, including pain, stiffness and physical function [23]. It is widely used in the OA literature, has been shown to be more responsive than other measures of knee pain [24,25], demonstrates good construct validity, particularly for the pain and physical function domains [23,24] and has been found to be a stable and reliable postal survey tool [26]. While designed primarily for hip and knee OA, the WOMAC has been used previously in validation purposes of other instruments associated with the lower limb[27,28].

• The General Well Being Index (GWBI) is a measure that has been specifically designed to assess psychological distress rather than physical incapacitation [29]. Statements are rated on a scale from 1 (significant distress) to 4 (no distress). It has been used in numerous clinical and non patient based groups [30]. It has demonstrated good internal consistency [31] and high test re-test reliability [30] and has been specifically adapted and validated for use in England [32].

• The Hospital Anxiety and Depression Scale (HADS) [33] is a 14-item scale designed to detect anxiety and depression, independent of somatic symptoms. It consists of two 7-item subscales measuring depression and anxiety. A 4-point response scale (from 0, representing absence of symptoms, to 3, representing maximum symptomatology) is used, with possible scores for each subscale ranging from 0 to 21. Higher scores indicate higher levels of disorder.

The research was conducted in compliance with the Helsinki Declaration with institutional review and ethical approval granted by the Leeds West Ethics Review Board.

### Statistical analyses

Rasch analysis is an iterative procedure which assesses a number of measurement attributes, as well as the assumptions which underpin the model [34,35]. The Rasch model shows what should be expected in responses to items if measurement (at the metric level) is to be achieved [36]. The model can be extended to the polytomous case and the version used here is that developed by Masters [37]. As the model specifies what is needed to transform ordinal into interval level data, the heart of the procedure is the assessment of fit of data to the model's expectations. A variety of fit statistics determine if this is the case [38]. Generally non-significant deviations from the model expectations are expected for chi-square-based statistics, and within range (± 2.5) for residual fit statistics. Two summary fit statistics for items and persons have an expected mean of zero, and standard deviation of one when data have perfect fit to the model [34].

Other aspects of Rasch analysis are concerned with testing model assumptions (such as local dependency and unidimensionality) and with the investigation of other attributes such as appropriate category response structure (for polytomous items) and for item bias, or differential item functioning (DIF) [39]. Full details of these procedures are given elsewhere [34,35]. The software used was RUMM2020 for the Rasch analysis [40], and SPSS Version 12.0 for other analyses.

For the Rasch analysis, a sample size of 150 patients is sufficient to estimate item difficulty to within ± 0.5 logits, with α of 0.01, and β of 0.2 [41]. This sample size is also sufficient, with the same power, to test for DIF where a difference of 0.5 standard deviations within the residuals can be detected for any two groups. It is important to note that Rasch analysis is distribution free, and does not require a 'representative' sample, but rather needs a good spread of respondents across the construct to be measured.

## Results

### Descriptive statistics

Of 635 questionnaires sent out, 390 were returned, a response rate of 61.4%. Of those returned, 65% completed the questionnaire, while 35% replied that they would not like to participate. This gave 259 valid respondents, well above the minimum sample size requirements. Initial analysis was undertaken in order to explore the potential of any responder bias and found that there were no differences in age or gender between responders and non-responders. The majority of the respondents were females (68.7%), with a mean age of 66.49 years (range: 21 to 98, SD 12.5 yrs) and mean disease duration of 12.6 years (range: 6 months to 45 years; SD 9.1 yrs). Almost one quarter of the sample was in paid employment. While the knee was the most common site of pain (40.2%), this was followed closely by the hand (39.8%), the foot (28.6%) and hip (23.9%). Multiple joint involvement was common, with the median number of joints affected being four. Only 11% of respondents reported only one site.

### Rasch analysis

Rasch analysis was conducted for each of the individual subscales of the PIPP with separate analyses reported for the PIPP Impact and PIPP Distress subscales. In the previous validation of the PIPP [18] a global recoding was conducted across all items to resolve disordered thresholds. This resulted in adequate PSI values (above .7) but these values were less than optimal for use at the individual level (requiring values above .85). In the current study, disordered thresholds were not rescored where overall model fit was achieved [42]. If misfit was observed, then individual rescoring was attempted and retained if fit improved. Marginally disordered thresholds were left unchanged.

The overall fit statistics for each subscale are presented in Table 1. Both the Impact and Distress Self-care scales showed good model fit and excellent person separation, with no misfitting items or DIF for sex, age or duration of disease. The overall fit statistics for the Impact Mobility subscale initially suggested some misfit to the Rasch model, however removal of item 11 (*ability to use a vehicle*) resulted in good fit to the model, with strong person separation reliability. No DIF was detected for gender or age. In the PIPP Distress Mobility subscale, item 12 (*ability to move around and within your house*) was removed resulting in adequate fit to the model (after Bonferonni adjustment), good person separation, and no further misfitting items and DIF.

**Table 1:** Final fit statistics for each PIPP subscale

**Analysis**	**No. items**	**Item Fit** **Residual**		**Person Fit Residual**		**Chi-Square Interaction**		**Person Separation PSI**
	
		*Mean*	*SD*	*Mean*	*SD*	*Chi-square (df)*	*p*	
*Impact*								
Self care	4	0.052	1.108	-0.398	1.103	9.305 (8)	0.317	0.932
Mobility	4	0.147	1.67	-0.378	.935	13.82(8)	0.086	0.92
Participation	4	0.285	0.510	-0.437	1.065	11.495 (8)	0.175	0.899
Relationships	4	0.004	1.156	-0.479	1.221	14.769(8)	0.064	0.955
Psychological	5	-0.164	0.388	-0.491	1.186	11.474(10)	0.32	0.900
*Distress*								
Self care	4	-0.205	1.15	-0.498	1.033	12.13(8)	0.145	0.92
Mobility	4	0.182	0.634	-0.366	0.982	13.381(8)	0.099	0.884
Participation	5	0.252	1.402	-0.507	1.255	20.755(10)	0.023	0.936
Relationships	4	0.037	1.059	-0.399	1.051	11.163(8)	0.193	0.956
Psychological	5	-0.106	0.320	-0.538	1.195	6.731(10)	0.751	0.918
								
*Ideal Value*		*0.000*	*1.000*	*0.000*	*1.000*		*>0.05*	*>0.7*

It was necessary to remove item 14 (*your ability to participate in family activities*) from the Impact Participation subscale in order to achieve satisfactory fit. No DIF was detected for gender, but two items in the Impact Participation subscale (item 15, 16) recorded DIF for age with item 16 showing higher probabilities for the under 67 years group and item 15 showing higher probabilities for the older age group (68 + years). The two items appear to be acting in opposite directions with respect to age, suggesting no bias at the subscale level. This was confirmed by comparing the person estimates derived from all items, with those derived from only DIF free items. The estimates at the individual person level were found to differ by less than 0.3 of a logit. It was therefore decided to retain both items in the Impact Participation subscale. The Distress Participation subscale recorded no evidence of DIF and showed overall model fit, with good person separation reliability, and no misfitting items.

The two Relationships subscales achieved good overall model fit, with high person separation reliability and no misfitting items. The targeting map for both subscales showed a skewed distribution with a floor effect indicating that a substantial proportion of the sample experienced very little impact of their health problem on relationships. The PIPP Impact Psychological Wellbeing scale initially showed good fit to the model; however two items (item 2: *Your moods and feelings*, item 23: *Your reliance on others for help*) showed DIF for age with item 2 showing higher probabilities for the under 67 yrs group and item 23 showing higher probabilities for the older age group (68 + years). Again no significant differences were found in the magnitude of person estimates derived from all items, compared to those without DIF. One item in the Distress Psychological Wellbeing subscale (item 23) also showed significant DIF for age, but comparison of person estimates showed little difference, therefore all items were retained.

After achieving fit to the Rasch model for all PIPP scales further testing was conducted to ensure unidimensionality. Independent t-tests compared person estimates derived from subsets of items identified from Principal Components Analysis of the residuals. All PIPP scales met the criteria for unidimensionality, with no more than 5% of *t*-values exceeding ± 1.96.

### Validation of PIPP subscales

The linear Rasch derived person estimates for each subscale were exported from RUMM2020 to SPSS. Among the Impact subscales (shown in the lower section of Table 2) the strongest correlation was between the Mobility and Participation scales (rho = .86) while the lowest was between the Self care and Relationship scales (rho = .53). A similar pattern of correlations were also observed among the Distress scales (see upper section of Table 2) with values ranging from .57 (Self Care and Relationships) to .90 (Mobility and Participation). This suggests that health problems that impact on an individual's mobility are also likely to have a substantial impact on their ability to work and to participate in family and community activities.

**Table 2:** Spearman correlations among Rasch derived PIPP subscale scores

	**Self care**	**Mobility**	**Relationships**	**Participation**	**Psychological**
**Self care**	**.93**	.*69*	.*57*	.*69*	.*70*
**Mobility**	.65	**.85**	.*59*	.*90*	.*87*
**Relationships**	.53	.60	**.96**	.*60*	.*66*
**Participation**	.60	.86	.57	**.88**	.*87*
**Psychological**	.66	.85	.67	.85	**.92**

Correlations among distress subscales are shown in italics in the top right diagonal of the table.

Correlations among impact subscales are shown in the bottom left diagonal of the table.

Correlations between corresponding impact and distress subscales are shown in bold on the diagonal.

Pairwise deletion of missing data: Ns ranged from 153 to 219.

All correlations significant at p < .001.

The correlations between the corresponding Impact and Distress scales are shown on the diagonal in Table 2. The strongest correlation was between the Relationship impact and distress subscales (rho = .96) with only slightly lower values for the other scales. These uniformly strong inter-correlations suggest a strong overlap between the ratings of the impact of a health problem and the distress that this causes. With up to 92% shared variance this is indicative of redundancy, suggesting the removal of one of the sets of scales. This was explored further by assessing the predictive ability of the PIPP scales against other validated measures.

#### Correlations with other measures

Consistent with expectations, there were strong correlations between the PIPP and WOMAC (see Table 3). The pain and physical function subscales of the WOMAC correlated strongly with the Mobility and Participation subscales of the PIPP. These results suggest that respondents with high levels of pain and disability as measured by the WOMAC, report substantial impact and distress on the various aspects assessed by the PIPP scales. In particular, pain and disability had the greatest impact on respondents' levels of mobility and their ability to participate in family and social activities.

**Table 3:** Spearman correlations between Rasch derived PIPP subscale scores, WOMAC, HADS and General Wellbeing

	WOMAC Pain	WOMAC Disability	HADS Depression	HADS Anxiety	General Wellbeing
**Impact**					
Self-care	.49	.59	.48	.45	-.55
Mobility	.68	.73	.63	.50	-.61
Relationship	.36	.49	.55	.44	-.52
Participation	.66	.71	.59	.43	-.52
Psychological	.65	.70	.71	.60	-.69

**Distress**					
Self-care	.52	.58	.50	.42	-.54
Mobility	.62	.68	.60	.55	-.62
Relationship	.39	.50	.50	.44	-.54
Participation	.69	.73	.62	.53	-.59
Psychological	.64	.67	.68	.65	-.73

Pairwise deletion of missing data: Ns ranged from 131 to 195.

All correlations significant at p < .001.

Inspection of the correlation matrix (see Table 3) also showed strong correlations between the two PIPP Psychological Wellbeing scales (impact and distress) and HADS Depression (rho = .71, rho = .68), the HADS Anxiety scale (rho = .60, rho = .65), and the General Wellbeing Index (rho = -.69, rho = -.73). Respondents reporting high impact and distress caused by their osteoarthritis also recorded higher levels of anxiety and depression, and lower levels of general wellbeing.

The equivalent strength of the observed relationships for the PIPP Impact and the Distress scales support the earlier suggestion of redundancy. Each of the pairs of correlations were of similar strength with the WOMAC, HADS and General Wellbeing Index. The Distress subscales appear to offer no further explanatory value over that provided by the Impact subscales.

#### Number of pain sites

In order to test the capacity of the PIPP to discriminate between two groups of patients who would be expected to demonstrate differences in their PIPP responses, the impact and distress of OA at several sites was explored. The number of pain sites recorded for each participant ranged from 1 to 14 (mean = 4.7, SD = 3.0, median = 4). A median split was utilized to create two groups (1 to 4 sites versus 5 to 14 sites). Mann-Whitney U tests comparing PIPP subscale scores for the two groups (see Table 4) showed statistically significant differences on all PIPP scales. In support of the validity of the PIPP, individuals with multiple pain sites reported higher levels of impact and distress across the aspects assessed by the PIPP.

**Table 4:** Comparison of Rasch derived PIPP subscale scores for low versus high numbers of pain sites

	**1 to 4 sites**		**5 to 14 sites**				
	*N*	*Median*	*N*	*Median*	*Mann-Whitney U*	*Z*	*p*

**Impact**							
Self-care	93	-3.39	93	-2.67	3442	-2.50	.012
Mobility	103	-1.36	98	-.25	3544	-3.65	.000
Relationship	93	-3.39	92	-2.28	3481	-2.30	.022
Participation	101	-.69	97	-.037	3318	-3.92	.000
Psychological	104	-1.04	98	-.25	3518	-3.80	.000
							
**Distress**							
Self-care	71	-3.13	84	-2.00	2436	-2.06	.039
Mobility	83	-1.00	89	-.30	2617	-3.30	.001
Relationship	71	-3.39	81	-2.51	2227	-2.51	.012
Participation	86	-1.12	86	-.22	2797	-2.76	.006
Psychological	87	-1.18	85	-.26	2444	-3.84	.000

Median values are Rasch derived person estimates ranging from -4.2 to +9.1 with high positive scores indicating higher impact or distress.

## Discussion

The aim of this study was to assess the psychometric properties of the PIPP scales using Rasch analysis and to investigate the impact and distress of OA from the patients' perspective. The PIPP subscales showed good fit to the Rasch model after minor adjustments to some scales. In the PIPP Impact Mobility subscale, item 11 (*ability to use a vehicle*) was identified for removal based on both empirical and qualitative grounds. Feedback from respondents suggested some confusion as to whether the item refers to the ability to use a vehicle as driver or passenger. After removal of this item all subscales showed high levels of person separation reliability, at levels (above .9) suitable for assessment at both the group and individual levels.

Validity testing provided support for the use of the PIPP in this OA sample. The scales showed strong and appropriate correlations with the WOMAC – participants with high levels of pain and disability as measured by the WOMAC reported high levels of impact and distress on the PIPP. Both PIPP Psychological Wellbeing scales correlated strongly with the HADS and the General Wellbeing Index. The strength of these relationships suggest that the PIPP could be used as a short assessment tool for several key constructs, reducing the need for substantial batteries of questionnaires when this may be contextually inappropriate or too time consuming.

The PIPP was originally designed to assess two related but distinct aspects of a health condition – impact and distress [18]. The results of the current study indicate that there is a very strong correlation between the two sets of scales and that they correlate in a very consistent manner with other validated measures. The distress subscales fail to add anything to the predictive power of the impact scales, suggesting that respondents were judging impact in relation to the amount of distress experienced. Therefore in future studies it is recommended that only the Impact subscales be administered, shortening the scale and reducing the load on respondents.

The study had a number of weaknesses. While the number of respondents returning questionnaires exceeded the minimum sample size requirements, the skewed scores of some subscales indicated the lack of uniformity of sample distribution which would have given the greatest degree of precision for item and person estimates. This was particularly so for the Relationships subscale and further work is needed to strengthen the evidence to support the validity of this scale. Again while the sample was large enough for this initial validation in OA, a much higher response rate, including higher agreement to participate amongst those returning a questionnaire, would have given the opportunity to have a 'set aside' sample to independently validate the revised subscales.

In the current study the external construct validation of the PIPP was tested against the pain and function subscales of the WOMAC. Although widely used in both research and clinical contexts, some studies using Rasch analysis have raised some concerns about the dimensionality, item fit and psychometric properties of the WOMAC [43,44]. Further research is needed using other well validated measures to confirm these findings.

The study also has a number of strengths. The sample represented a wide range of joint involvement making the work generalisable for OA. The Rasch measurement model is state-of-the-art in measurement science, placing the most rigorous demands upon data quality, satisfying the basic axioms for constructing interval scale measurement [45].

## Conclusion

In conclusion, measuring the impact of OA from the perspective of the bio-psychosocial model provides important information on the wider patient-perceived impact of the disease, rather than focussing on impairment and activity limitation. As such, the PIPP provides a different perspective to existing outcome measures used in OA, and offers a multi-domain capability for examining the complex inter-relationships that exist within the bio-psychosocial model. Given this sample included several different locations of OA and multiple joint presentation, the shortened version, using only the impact scales of PIPP, demonstrated good internal construct validity for use in a wide range of OA presentations.

## List of abbreviations

OA: Osteoarthritis; PIPP: Perceived Impact of Problem Profile; ICF: International Classification of Functioning, Disability and Health; WOMAC: The Western Ontario McMasters University Osteoarthritis Index; GWBI: General Well Being Index; HADS: Hospital and Anxiety Scale; DIF: Differential Item Functioning; PSI: Person Separation Index.

## Competing interests

The authors declare that they have no competing interests.

## Authors' contributions

AMK, AT and PC designed the study, AMK collected the data, JP conducted the statistical analyses, RM participated in the analyses, and all authors participated in writing and reviewing of the manuscript.
